# The IOP lowering effects of “planning” selective laser trabeculoplasty in open angle glaucoma

**DOI:** 10.3389/fmed.2022.1013260

**Published:** 2022-10-06

**Authors:** Yi-Ching Chu, Pei-Yao Chang, Jia-Kang Wang, Tzu-Lun Huang, Yung-Ray Hsu

**Affiliations:** ^1^Department of Ophthalmology, Far Eastern Memorial Hospital, New Taipei City, Taiwan; ^2^Department of Medicine, National Taiwan University Hospital, Taipei, Taiwan; ^3^Department of Healthcare Administration, Asia University, Taichung, Taiwan; ^4^Department of Electrical Engineering, Yuan Ze University, Taoyuan City, Taiwan; ^5^School of Medicine, National Yang Ming Chiao Tung University, Hsinchu, Taiwan; ^6^Department of Healthcare Administration and Department of Nursing, Oriental Institute of Technology, New Taipei City, Taiwan

**Keywords:** selective laser trabeculoplasty, intraocular pressure, IOP fluctuation, adherence, open angle glaucoma

## Abstract

**Purpose:**

To investigate whether the planning of selective laser trabeculoplasty (SLT) influences the intraocular pressure (IOP) in patients with open angle glaucoma (OAG).

**Methods:**

In this retrospective case-control study conducted on patients with OAG who planned to undergo SLT treatment (SLT group) or a visual field examination (VF group), we collected the demographic data, IOP on the planning day and on the scheduled day of the SLT treatment or VF examination. ΔIOP was defined as the IOP change between the planning day and the scheduled day. We used multivariable regression analyses and linear mixed model to evaluate the association between the abovementioned factors and ΔIOP in the VF group and the treatment eye (SLT_t_) and fellow eye (SLT_f_) of the SLT group.

**Results:**

One hundred and fifty-three eyes of 102 patients with OAG were included, of which 51 patients in the SLT group and 51 patients in the VF group. The ΔIOP was −1.92 ± 2.77 mmHg in the SLT_t_, −0.65 ± 2.47 mmHg in the SLT_f_ and −0.08 ± 1.73 mmHg in the VF group (*P* < 0.05). Both multivariable regression analysis between the VF and SLT_t_ group and linear mixed model in the SLT group showed significant negative association between the ΔIOP and SLT arrangement (*P* < 0.05). There was no significant association between ΔIOP and age, gender, baseline IOP, IOP fluctuation, nor SE.

**Conclusions:**

The IOP was significantly reduced in patients with OAG after “planning” of SLT treatment, even without actual performing the laser treatment in our retrospective case-control study.

## Background

Intraocular pressure (IOP), a major risk factor for glaucoma progression, was well known that it was not fixed but a fluctuated value over time. The IOP fluctuation could be categorized according to the period 1. Studies on IOP fluctuation defined the IOP variation occurring within a 24-h period as diurnal or short-term IOP fluctuation (1–5). Short-term IOP fluctuation also referred to IOP variation that occurred within a day or over days to weeks (1–5) while long-term was that through months to years (1, 5–7).

Selective laser trabeculoplasty (SLT) has selective effect on melanotic elements within the trabecular meshwork, facilitating flow into Schlemm's canal and then subsequent reduction in IOP (8). The IOP lowering effect of SLT was about 11–40% reduction in OAG (9, 10) and angle closure glaucoma (ACG) (11–14). In eyes with OAG or ACG, in terms of IOP control, primary treatment with SLT was comparable or even better than traditional treatment with topical prostaglandin analog medications (15, 16). Most studies regarding the SLT treatment were focused on the effect on IOP reduction, but there were limited studies designed to find the association of IOP fluctuation and the arrangement of SLT.

The IOP fluctuation presented higher in patients with glaucoma and abundant studies reported that IOP fluctuation may be related to the risk of glaucoma development and progression (6, 7, 17). The Los Angeles Eye Study revealed that IOP fluctuations were associated with OAG risk when IOP < 15 mmHg (7). Asrani et al. reported that diurnal IOP fluctuation in well controlled glaucoma patients was a risk factor for disease progression (18). A *post-hoc* study conducted by Nouri using the patients in the Advanced Glaucoma Intervention study (AGIS) showed that IOP fluctuation between visits was an independent risk factor for glaucoma progression (19).

Numerous studies aimed to evaluate the factors associated with IOP fluctuation, such as baseline IOP (20), blood pressure (21), medications use (22), postural change (23), or exercise (24). Few studies were designed to evaluate the association of IOP fluctuation with the arrangement of an examination or intervention. Therefore, we performed a retrospective study to investigate whether the planning of selective laser trabeculoplasty influences the IOP in OAG patients.

## Materials and methods

The protocol of the study, which followed the principles of the Declaration of Helsinki, was approved by the Institutional Review Board of Far Eastern Memorial Hospital (FEMH) in Taiwan (109108-E).

The SLT group included the patients with OAG who were scheduled to receive SLT treatment between January 2018 and March 2019. Bilateral eyes of the patients in the SLT group were divided into two groups, the treatment eye (SLT_t_) and the fellow eye (SLT_f_). The treatment eye was the eye scheduled to receive the SLT treatment. If the patient received SLT treatment of both eyes during this period, only the first eye received SLT treatment was included as the treatment eye. The VF group included the patients who visited the glaucoma clinic in FEMH between January and Dec 2019, and the worse eye was chosen for the statistical analysis. Patients were 20 years or older, visual acuity of 20/40 or better, and no previous intraocular surgery, except uncomplicated cataract surgery 6 months before entering the trial.

The exclusion criteria were eyes with ocular trauma, macular disease, or other optic nerve disease; those whose corneal pathology or anterior chamber pathology which obscured gonioscopic view to the angle and fundoscopic exam. Additionally, patients who used steroid eye drops within 3 months of the SLT treatment or VF examination were also excluded.

Age, gender, mean deviation (MD) of the visual field examination, central corneal thickness (CCT), spherical equivalent (SE), IOP, and the types and bottles of anti-glaucoma medications were collected in both groups. Noncontact pneumotonometry (Tonopachy^TM^ NT-530P, NIDEK, Japan) was used for IOP measurement. We collected the IOP on the scheduled day of the SLT treatment or VF examination on the planning day and the two previous visits. Baseline IOP and IOP fluctuation was defined as the mean and standard deviation of the three IOP measurements before the scheduled day. The change of IOP (ΔIOP) was defined as the IOP of the scheduled day minus the IOP of the planning day.

### Statistical analysis

Statistical analysis was performed using SPSS ver. 22.0 (SPSS Inc., Chicago, IL, USA). To evaluate the difference between the SLT group and the VF group, we compared the treatment eyes of the SLT group (SLT_t_) and the VF group. Student *t*-test and the Mann-Whitney test were used to compare the differences of the following continuous variables between the two groups: age, CCT, SE, MD, baseline IOP, IOP fluctuation, and ΔIOP based on the distribution of the variables. Simple linear regression analysis was used to evaluate the association between the above factors and ΔIOP. If the factor had significant correlation with ΔIOP or significant difference between two groups, multivariable linear regression would be performed for further analysis. *P* < 0.05 was considered statistically significant.

To evaluate the difference between each eye in the SLT group, paired *t*-test and Wilcoxon sign-rank test were performed to compare the differences between the treatment eye and the fellow eyes in the SLT group. Linear mixed model was used to compare IOP changes in the SLT group between both eyes.

## Results

One hundred and fifty-three eyes of 102 patients with OAG were included in this study, of which 51 people were in the SLT group and 51 people were in the VF group.

The mean age of the patients was 56.21 ± 14.05 in the SLT group and 45.08 ± 12.40 the VF group (*P* < 0.05). The visual field defect was significantly worse in the treatment eye of the SLT group (SLT_t_) than the VF group (−13.68 ± 8.94 dB and −4.78 ± 6.15 dB, *P* < 0.05).

In the SLT_t_ group, the IOP on the planning day was 15.88 ± 3.05 mmHg and the IOP on the scheduled day was 17.80 ± 2.56 mmHg. In the VF group, the IOP on the planning day was 16.68 ± 3.08 mmHg and the IOP on the scheduled day was 16.76 ± 3.31 mmHg. The baseline IOP was 17.27 ± 3.21 mmHg in the SLT_t_ and 16.98 ± 3.09 mmHg in the VF group. IOP fluctuation was 2.53 ± 1.79 mmHg in the SLT_t_ and 1.61 ± 1.12 mmHg in the VF group (*P* < 0.001). The ΔIOP was −1.92 ± 2.77 mmHg in the SLT_t_ group and −0.08 ± 1.73 mmHg in the VF group (*P* < 0.001). The patients in the SLT group received significantly more amounts of glaucoma medications (1.86 ± 0.60 mmHg in the SLT_t_, and 1.31 ± 0.55 mmHg in the VF group, *P* < 0.001), more carbonic anhydrase inhibitors and prostaglandin analogs for treatments (Table 1).

**Table 1:** Patients' characteristics in the SLT and VF group.

	**SLT group (*****n*** = **102)**			**VF group (*n* = 51)**	**P_2_**
	**Treatment eye (*n* = 51)**	**Fellow eye (*n* = 51)**	**P_1_**		
Age (years)	56.21 ± 14.05			45.08 ± 12.40	< 0.001
Gender (male, %)	37 (72.5%)			30 (58.8%)	0.144
SE (D)	−4.66 ± 3.98	−4.03 ± 5.76	0.31	−5.94 ± 3.13	0.08
CCT (mm)	552.47 ± 30.08	551.88 ± 28.98	0.74	561.49 ± 37.05	0.18
MD (dB)	−13.68 ± 8.94	−7.84 ± 7.88*	< 0.001	−4.78 ± 6.15*	< 0.001
Baseline IOP (mmHg)	17.27 ± 3.21	15.59 ± 2.45*	< 0.001	16.98 ± 3.09	0.64
IOP fluctuation (mmHg)	2.53 ± 1.79	1.83 ± 0.94*	< 0.001	1.61 ± 1.12*	< 0.001
ΔIOP (mmHg)	−1.92 ± 2.77	−0.65 ± 2.47*	< 0.001	−0.08 ± 1.73*	< 0.001
Average medication bottle amount	1.86 ± 0.60	1.67 ± 0.74	0.171	1.31 ± 0.55*	< 0.001
**Medication type**
α-agonist (*n*, %)	19 (37.3)	15 (29.4)	0.264	13 (27.7)	0.143
β-blocker (*n*, %)	43 (84.3)	38 (74.5)	0.164	34 (66.7)	0.038
CAI (*n*, %)	28 (54.9)	23 (45.1)	0.214	13 (25.5)	0.002
PG (*n*, %)	39 (76.5)	36 (70.6)	0.327	26 (51.0)	0.01

CAI, carbonic anhydrase inhibitor; CCT, central corneal thickness; MD, mean deviation; IOP, intraocular pressure; ΔIOP, IOP changes; PG, prostaglandin analogs; SE, Spherical equivalent; SLT, Selective laser trabeculoplasty; VF, Visual Field test.

P_1_= *P* value between treatment eye and fellow eye of the SLT group.

P_2_= *P* value between treatment eye of the SLT group and the VF group.

*P < 0.05 significance.

Univariable analysis through simple linear regression model revealed that the following factors had significant association with ΔIOP: SE (*b* = −0.293, *P* = 0.003), IOP fluctuation (*b* = −0.387, *P* < 0.001), baseline IOP (*b* = −0.221, *P* = 0.026), SLT arrangement (*b* = −0.373, *P* < 0.001). Multivariable linear regression model showed only SLT arrangement (*b* = −0.268, *P* = 0.009) has significant negative association with ΔIOP between the scheduled day and the planning day. There was no significant association between ΔIOP and age, SE, baseline IOP, nor IOP fluctuation was found through multivariable analysis (Table 2).

**Table 2:** Correlation between selected variables and ΔIOP (In SLT vs. VF group).

	**Univariable linear regression analysis**		**Multivariable linear regression analysis**	
	**Coefficient (β)**	***P*-value**	**Coefficient (β)**	***P*-value**
Age	−0.278	0.005*	−0.029	0.818
Gender	0.042	0.676		
SE	−0.293	0.003*	−0.193	0.095
CCT	0.109	0.273		
MD	−0.112	0.26		
SLT	−0.373*	< 0.001	−0.268	0.009*
Baseline IOP	−0.221*	0.026	−0.162	0.121
IOP fluctuation	−0.387*	< 0.001	−0.186	0.090
Med bottles	−0.049	0.625		

CCT, central corneal thickness; IOP, intraocular pressure; ΔIOP, IOP changes; MD, mean deviation; Med bottle, the total bottles of glaucoma medications; SE, Spherical equivalent; SLT, Selective laser trabeculoplasty; V, Visual Field test.

*P < 0.05 significance.

Within the two eyes of the patient receiving SLT, the visual field test revealed significantly worse VF in the treatment eye than the fellow eye (MD = −13.68 ± 8.94 dB and −7.84 ± 7.88, *P* < 0.001). The baseline IOP and IOP fluctuation was 15.59 ± 2.45 and 1.83 ± 0.94 in the SLT_f_ group, which was significantly lower than those of the SLT_t_ group (*p* < 0.05). The ΔIOP in the SLT_f_, was significantly different from that of SLT_t_ (−0.65 ± 2.47 mmHg and −1.92 ± 2.77 mmHg, *p* < 0.001). Linear mixed models with age, gender, baseline IOP, IOP fluctuation, MD and SLT arrangement revealed that SLT arrangement had significant negative influence on ΔIOP in the SLT group (Table 3).

**Table 3:** The influence of planning SLT treatment on ΔIOP in the SLT group.

**Covariate**	Δ**IOP**		
	**Coefficient (β)**	**95% CI**	***p*-value**
Age	−0.05	−0.10, 0.01	0.08
Male gender	−0.19	−1.38, 1.30	0.8
Baseline IOP	−0.15	−0.37, 0.07	0.18
IOP fluctuation	−0.22	−0.64, 0.20	0.30
MD	−0.02	−0.09, 0.04	0.51
SLT	−0.98	−1.83, −0.13	0.02*

MD, mean deviation; IOP, intraocular pressure; ΔIOP, IOP changes from the planning day to the scheduled day of SLT; SLT, Selective laser trabeculoplasty.

*P < 0.05 significance.

## Discussion

We compared the IOP change between the SLT_t_ group and the VF group to find the difference between the subjects who received SLT treatment and who did not. The both eyes of the SLT group (SLT_t_ and SLT_f_) were compared to search for the influence of planning SLT on IOP change. We found that the IOP was significantly reduced after planning of the SLT treatment. To the best of our knowledge, the current study was the first study that investigated the influence of planning SLT on IOP change in patients with OAG.

In the SLT_t_ group and VF group, the IOP change from the planning day to the scheduled day of intervention (ΔIOP) had significant association with age, SE, IOP fluctuation, baseline IOP and arrangement of SLT using univariable simple linear regression analysis. Multivariable linear regression model showed that only the arrangement of SLT had significant negative association with ΔIOP, which meant the IOP was significantly reduced in patients with OAG after “planning” of SLT treatment, even without actual performing the laser treatment. We assumed that compliance or adherence issues might be the most possible reason to account for this finding. Poor adherence to antiglaucoma medication has been reported to be related to higher IOP and IOP fluctuation (25–27). A randomized control study investigating refill adherence showed that newly prescribed medications in OAG patients with good adherence had less IOP fluctuation in 24 months follow-up (25). A cross-sectional study using Morisky medication adherence scale revealed a trend of increase in IOP with increase in the score of nonadherences (26). In a study evaluating the reasons for medication prescription, the physicians commented that persistent IOP elevation was often a sign of nonadherence (27). Nonadherence has been reported to be related to patients with multiple medications. Adherence reported by patient interviewing and chart review decreased from 81 to 50% with the amounts of medication increased (28). In a large retrospective study including more than 37,000 glaucoma patients, the persistence of medication through 1 year was decreased from the one-bottle group (35.3%) to the three-bottle group (23.9%) (29). In our study, the average amount of anti-glaucoma agents was 1.78 bottles in the SLT group, which was significant more than 1.31 bottles in the VF group. Because of more IOP lowering bottles, the adherence of medication may be less in the SLT group than in the VF group. We speculated that the arrangement of SLT, which is an invasive intervention for IOP control clearly delivered the message of progression of disease and poor control of the IOP to the patients. Therefore, the arrangement of SLT may increase the patients' adherence for antiglaucoma medication compared to those who were only arranged for a routine follow-up examination like VF examination.

Nakakura conducted a prospective study using Goldmann applanation tonometry revealed that the office IOP fluctuation in 6 months was 2.75 ± 1.68 mmHg in POAG patients using three kinds of antiglaucoma agents (30). Tojo et al. using Triggerfish^®^ contact lens sensor measured the 24-h IOP fluctuation and found positive correlation of short term IOP fluctuation (24-h fluctuation) and long term IOP fluctuation (5). It was believed that IOP fluctuation had significant correlation with mean IOP (6, 20, 28, 29), which means patients with higher baseline IOP usually had larger IOP fluctuation. In our study, we also found significant positive correlation between IOP fluctuation and the mean IOP of the three visits before the scheduled SLT treatment. Comparing bilateral eyes of the patients in the SLT group, the ΔIOP were negative values which means the IOP decreased at the scheduled day from the planning day of SLT treatment in bilateral eyes. The value of ΔIOP was more in the SLT_t_ than in the SLT_f_, and it may be related to the higher baseline IOP in the SLT_t_ group. On the other hand, the mean IOP and IOP fluctuation had significant correlation with the ΔIOP in univariable analysis. However, no significant correlation was found when the mean IOP and IOP fluctuation were put simultaneously in the multivariable analysis. It may be caused by the confounding effect of the significant positive correlation between baseline IOP and IOP fluctuation.

The average age of patients in the current study was 45.08 years old in the VF group and 56.21 years old in the SLT group. Numeral studies investigated the relation between age and adherence, but the results were controversial. In newly treated individuals with diagnosed OAG, greater adherence to medications was noted with increasing age (31). A retrospective chart review in the United Kingdom showed that glaucoma treatment adherence, which was defined as average difference in the actual number of prescriptions collected compared to 12 prescriptions required annually, improved with increasing age but may be related to the drop wastage in elderly patients (32). However, there was no significant correlations between age and adherence were found in a multicenter observational study in Korea (33) and in Taiwan (34). In our study, no significant correlation between the age and ΔIOP was noted in multivariable analysis. Although the mean age of the two groups were significant different in the current study, they were relatively younger and usually counted in the same age groups in other studies (32–34). Besides, the adherence of medication in age 40–49 was similar to that of age 50–59 in Nordstrom's study (31). There was no significant correlation in the age and ΔIOP may be related to the relatively younger age in our patients.

There were some limitations in our study. First, our study was a retrospective study, there was a significant difference in the age and glaucoma severity between the two groups. However, we found age and visual field defect had no significant correlation with the IOP change (ΔIOP). Second, the IOP had diurnal change which may influence the value of IOP change. In our study, the patients came to our clinics regularly in the morning or in the afternoon for IOP measurement, which may decrease the influence of the diurnal IOP fluctuation. Third, we did not directly evaluate the adherence of our patients due to our retrospective design. We could not provide direct evidence of the relationship of the IOP and the adherence.

In summary, we found that the arrangement of SLT had significant negative association with the change of the IOP in this retrospective case-control study. Additional prospective studies investigating whether the arrangement of intervention such as laser treatment or even filtering surgery influence the IOP and the adherence to glaucoma medications in the long-term follow-up are needed.

## Value statement

### What was known

Poor adherence to topical antiglaucoma medication was associated with higher IOP fluctuation.

### What this study adds

The arrangement of selective laser trabeculoplasty (SLT) even without performing it lowered the IOP in the patients with open angle glaucoma (OAG).

## Data availability statement

The original contributions presented in the study are included in the article/supplementary material, further inquiries can be directed to the corresponding author.

## Ethics statement

The protocol of the study was approved by the Institutional Review Board of Far Eastern Memorial Hospital (FEMH) in Taiwan (109108-E). Informed consent was waived by the IRB due to retrospective design of the study.

## Author contributions

Y-CC and P-YC designed the study, collected the clinical data, performed the statistics, wrote the main manuscript text, and prepared tables. J-KW, T-LH, Y-RH, and P-YC reviewed, corrected, and approved the manuscript. All authors contributed to the article and approved the submitted version.

## Funding

This project was supported by a grant obtained from the Far Eastern Memorial Hospital (FEMH-2022-C-060).

## Conflict of interest

The authors declare that the research was conducted in the absence of any commercial or financial relationships that could be construed as a potential conflict of interest.

## Publisher's note

All claims expressed in this article are solely those of the authors and do not necessarily represent those of their affiliated organizations, or those of the publisher, the editors and the reviewers. Any product that may be evaluated in this article, or claim that may be made by its manufacturer, is not guaranteed or endorsed by the publisher.
